# Current clinical applications of RNA-LNPs in cancer: a promising horizon for targeted therapies

**DOI:** 10.17179/excli2025-8132

**Published:** 2025-02-27

**Authors:** Md Sadique Hussain, Gyas Khan

**Affiliations:** 1 Uttaranchal Institute of Pharmaceutical Sciences, Uttaranchal University, Prem Nagar, Dehradun 248007, Uttarakhand, India; 2 Department of Pharmacology and Toxicology, College of Pharmacy, Jazan University, Jazan 45142, Saudi Arabia

## ⁯⁯⁯

In the past few decades, the term 'messenger RNA' or mRNA has come to play a significant role in the biotechnology and medical fields. Previously considered far too immunogenic and unstable to serve as a therapeutic entity, mRNA is now on the rise, thanks to advancement in delivery systems and molecular stabilization. Pandora has been created for the mRNA vaccine, especially for COVID-19, meaning that the general public has expressed new interest in mRNA-based pharmaceuticals. Starting with vaccine technology and progressing to gene therapies and protein replacement strategies, mRNA is revealing itself as a platform for treating various medical conditions. One of the most dynamic and promising is cancer therapy, which employs lipid nanoparticles (LNPs) to deliver mRNA therapy to tumor or immune cells. With this letter, the author aims to explore the current clinical applications of RNA-LNPs in cancer, their opportunities and limitations, and the potential future implications to elucidate this paradigm shift in cancer treatment.

LNPs help with the delivery of mRNA by encapsulating and protecting mRNA in charge of its stability and promote its subsequent uptake and the release of its contents into the cytoplasm. However, it is significant to remember that by enhancing the stability of these molecules and avoiding their degradation LNPs have a tremendous input in the success of mRNA therapies. Besides, stability is not the sole strength of the LNPs; they can also be engineered to deliver certain therapies to targeted tissues or immune cells, making them suitable for cancer applications (Eygeris et al., 2022[3]; Oberli et al., 2017[7]).

The potential uses of the mRNA-loaded LNPs in cancer therapy were studied in various* in vitro*, *in vivo*, and clinical studies. It is in immunotherapy that the main aim of these therapies is to trigger an immune response, to even more selectively target the cancer cells, and in some instances, manage to change the tumor milieu (Dana et al., 2020[2]).

Another significant application of mRNA-LNPs for cancer therapy is the field of mRNA vaccine methodologies. These vaccines are designed to generate an encoded tumor antigen by which the immune system shall be able to locate and destroy the cancer cells. Several cancer types such as melanoma, lymphoma, and hepatocellular carcinoma, have been explored in preclinical studies using mRNA-LNP-based vaccines. For example, in melanoma, vaccines based on mRNA have been used to encode tumor antigens MART1 and LAMP1, stimulating immune responses and increasing survival rates in mouse models (Perche et al., 2011[8]).

Like the application above, non-Hodgkin's lymphoma mRNA-based LNPs have been employed where LNP-lymphocyte targeted delivery involves the spleen and lysing glands (Fan et al., 2018[4]). This approach can trigger an immune response that will help fight the cancer. In these models, mRNA-based vaccines have been demonstrated to elicit CD8+ T cells, which play a central role in cancer cell destruction.

One of the most specific analyses was performed using mRNA-lipid formulations of modified OVA mRNA. Such formulations elicited robust CD8+ T-cell responses in melanoma models and showed the potential how to optimize mRNA vaccines to enhance immunogenicity through alteration of the lipid profiles (Oberli et al., 2017[7]). However, the inclusion of immune adjuvants such as galactosylceramide further improved tumor control, significantly reducing B16-OVA melanoma tumor growth (Verbeke et al., 2019[12]).

In addition to cancer vaccines, therapeutic mRNAs designed to intervene with certain molecular pathways in tumor development, can also be 'packed' into mRNA-LNPs. For example, the use of polymer-lipid hybrid nanoparticles carrying modified PTEN mRNA arrested the progression of prostate cancer by suppressing the cell survival signaling cascade PI3K/AKT and stimulating programmed cell death (Wise et al., 2017[14]). In the same way, in hepatocellular carcinoma, the mRNA delivery through LNP has cut the size of the tumor and prolonged the lifespan of transgenic mice having overexpression of MYC oncogenes, thus, showing the capability of mRNA-LNP for delivering effective cancer therapies with minimal side effects on normal cells (Lai et al., 2018[5]).

One more field, where RNA-LNPs demonstrate their potential, is the therapy of breast cancer. Cationic lipid-mRNA systems have been compared to synthetic antibodies like trastuzumab (Herceptin), revealing superior biodistribution efficiency for mRNA delivery systems. In a study using HER2+ mice, mRNA coding trastuzumab yielded higher serum concentration and better tumor inhibition than Herceptin, with no apparent toxicity in the treated mice (Rybakova et al., 2019[11]). This shows that mRNA-based therapeutics are possibly safer and more efficient means of treatment than the typical monoclonal antibody treatment.

### Preclinical and clinical evidence for cancer immunotherapies

Recent data from preclinical and phase I and II clinical trials have confirmed the applicability of mRNA-LNP therapy in cancer immunology. For instance, a study by Silva and his colleagues explored three different mRNA formulations targeting HPV-16-related tumors in mice. These formulations encapsulated in LNPs had a high intensity of E7-specific CD8+ T cells to eliminate subcutaneous tumors and prevent the recurrence. These mRNA vaccines have shown superiority over DNA and protein vaccines, highlighting to their ability to revolutionize cancer immunotherapy (Ramos da Silva et al., 2023[10]).

One notable advancement is creation of an endogenous LNP targeting the lymph nodes, called 113-O12B, which enhanced CD8+ T cell activity, and tumor suppression in addition to immune memory in treated mice (Chen et al., 2022[1]). This investigation also demonstrates that, unlike most conventional cancer therapies that solely aim at attack cancer cells, LNPs can reshape the anti-tumor immune microenvironment, thereby providing a more sustained approach to cancer treatment.

### Challenges in RNA-LNP cancer therapies

Despite a wide range of possibilities in cancer treatment by RNA-LNPs, important issues that must be solved before their widespread clinical application. A major challenge is related to the delivery of the interventions, in terms of showing that they are effective and can reach the right audience. Although measurements made by LNPs can be adjusted to deliver targeted tissues, the delivery to tumor cells often harms other benign cells. In addition, immune responses elicited by LNPs themselves can result in adverse inflammatory reactions, and adjusting the lipid content of the nanoparticle to reduce such reactions is still under investigation (Landesman-Milo and Peer, 2012[6]).

Another challenge lies in maintaining the stability of the mRNA payload: this must be effected while maintaining a reasonably small size for the construct as a whole. The problem with LNP formulation is that despite the continual improvement in LNP formulations mRNA remains inherently unstable thereby posing the challenge of making sure that mRNA remains stable enough to be delivered effectively to the tumor cells (Ramadan et al., 2024[9]). Furthermore, manufacturing RNA-LNPs still has a high cost for large-scale production and clinical practice, which could also be a problem (Webb et al., 2022[13]).

### Future perspectives and directions

Looking ahead, the future of RNA-LNP-based therapies in cancer is promising, but several areas need further exploration. With genomic sequencing and tumor characterization becoming increasingly sophisticated, RNA-LNPs might be employed to synthesize patient-specific therapy in the form of cancer vaccines that would target the individual antigens identified in each tumor. This could enhance the immune response and limit adverse effects on normal tissues. In addition, the combination of already printed mRNA-LNPs with other treatment regimens, including immune checkpoint inhibitors, oncolytic viruses, or targeted treatments, may increase the therapeutic effectiveness of cancer treatment. For instance, the use of mRNA-LNPs could apply immune-activating agents conjugate with checkpoint inhibitors to elicit a more effective anti-tumor activity. It is, therefore, expected that with an increasing number of clinical trials for mRNA-LNP therapies in cancer, there will be continued generation of real-world evidence, giving more light on the safety and effectiveness of these treatments.

### Conclusion

RNA-LNPs represent a major breakthrough in cancer therapy; RNA-LNPs are a powerful tool for immune systemic cytokines, treatment, and gene transfer. But there are still questions that need to be solved and we can see a positive trend in the improvement of the preclinical and clinical stages. As more knowledge and work are conducted regarding the advanced methods of delivering mRNA-based therapeutic agents may in the future become one of the prime modalities of cancer treatment and management. Therefore, the future of cancer treatment might well be in RNA modification, which opens a new page in precision oncology.

## Declaration

### Author contributions 

Md Sadique Hussain: Supervision, writing - original draft, review & editing. 

Gyas Khan: Conceptualization, investigation, formal analysis, software, visualization.

Both authors have approved the final version of the manuscript. 

### Conflict of interest

None.

### Declaration of Generative AI and AI-assisted technologies in the writing process

During the preparation of this work the author(s) used ChatGPT to correct the grammatical and typographical errors in the manuscript. All authors have read and approved the final version of the manuscript.
